# Phytoplankton Diversity and Community Composition along the Estuarine Gradient of a Temperate Macrotidal Ecosystem: Combined Morphological and Molecular Approaches

**DOI:** 10.1371/journal.pone.0094110

**Published:** 2014-04-09

**Authors:** Pauline Bazin, Fabien Jouenne, Thomas Friedl, Anne-Flore Deton-Cabanillas, Bertrand Le Roy, Benoît Véron

**Affiliations:** 1 Université de Caen Basse-Normandie, UMR BOREA “Biologie des Organismes et Ecosystèmes Aquatiques,” Caen, France; 2 Centre National de la Recherche Scientifique (CNRS), Institut Ecologie et Environnement (INEE), UMR BOREA, Caen, France; 3 Algobank-Caen, Université de Caen Basse-Normandie, Caen, France; 4 Department Experimentelle Phykologie und Sammlung für Algenkulturen (EPSAG), Georg-August-Universität Göttingen, Göttingen, Germany; University of Connecticut, United States of America

## Abstract

Microscopical and molecular analyses were used to investigate the diversity and spatial community structure of spring phytoplankton all along the estuarine gradient in a macrotidal ecosystem, the Baie des Veys (eastern English Channel). Taxa distribution at high tide in the water column appeared to be mainly driven by the tidal force which superimposed on the natural salinity gradient, resulting in a two-layer flow within the channel. Lowest taxa richness and abundance were found in the bay where *Teleaulax*-like cryptophytes dominated. A shift in species composition occurred towards the mouth of the river, with the diatom *Asterionellopsis glacialis* dramatically accumulating in the bottom waters of the upstream brackish reach. Small thalassiosiroid diatoms dominated the upper layer river community, where taxa richness was higher. Through the construction of partial 18S rDNA clone libraries, the microeukaryotic diversity was further explored for three samples selected along the surface salinity gradient (freshwater - brackish - marine). Clone libraries revealed a high diversity among heterotrophic and/or small-sized protists which were undetected by microscopy. Among them, a rich variety of Chrysophyceae and other lineages (e.g. novel marine stramenopiles) are reported here for the first time in this transition area. However, conventional microscopy remains more efficient in revealing the high diversity of phototrophic taxa, low in abundances but morphologically distinct, that is overlooked by the molecular approach. The differences between microscopical and molecular analyses and their limitations are discussed here, pointing out the complementarities of both approaches, for a thorough phytoplankton community description.

## Introduction

Estuaries form transition zones linking freshwater and marine biomes. Due to mixing of both distinct water bodies, they are characterized by pronounced gradients of physical and chemical components [1]. These factors strongly influence the phytoplankton community structure and other microbial eukaryotes along the resulting continuum. Major estuaries are usually classified into three types based on their longitudinal salinity distribution and flow characteristics: i) highly stratified or salt wedge, ii) partially mixed, or iii) well mixed [2]. However, for many systems wherein physical forces are highly variable, such as the shallow macrotidal estuaries [3], assignation to one estuary type considering temporal (seasonality, tidal cycle variation) and spatial (lower, intermediate, upper estuary) variations is difficult.

Protists are key components of aquatic food webs, both as major primary producers and as important consumers of bacteria in the “microbial loop” [4]. In recent years, the rise of molecular microbial ecology has opened the possibility of studying protist diversity independently of morphological considerations. Such molecular environmental surveys revealed a high diversity of eukaryotic lineages and contributed to our current understanding of microbial food web structure and biogeochemical processes in aquatic systems [5], [6]. This approach has been applied in a wide variety of ecosystems, including oceanic/coastal waters, freshwater ecosystems, and many extreme environments such as anoxic systems or deep-sea vents [7]–[10]. Most studies have focused on small size protists (<3–5 μm) which usually escape detection with traditional microscopy and are difficult to isolate. Insight of many novel eukaryotic lineages divergent from known protist sequences suggests that a large fraction of these communities still remains to be discovered [11].

Microbial communities inhabiting aquatic transition systems received relatively little attention in the past. Most investigations were based on morphological approaches and carried out in well-known and vast estuaries of the world, focusing generally on the large phytoplanktonic fractions [12], or restricted to a specific part of the estuary [13]. Very few diversity surveys using molecular techniques have been conducted so far on phytoplankton and other protists inhabiting rivers or marine–freshwater transition zones, except for some recent studies restricted to a single point and following temporal dynamics [14], [15].

The Vire River flows into the Baie des Veys which is located on the French coast, facing the English Channel. While much effort has been made to study temporal dynamics of phytoplankton and primary production in this macrotidal estuarine ecosystem [16]–[19], detailed studies of protistan diversity and their spatial patterns in such transitional waters are still lacking. Planktonic and benthic microalgae within the Baie des Veys have been mainly characterized using microscopy techniques [16], [17], and only one study has been conducted in the Vire River estuary, limited to a single station located in the lower zone [19].

In this context, we investigated here the diversity and spatial distribution of the spring phytoplankton community along the entire freshwater-to-marine continuum of the Vire River (Baie des Veys). This spatial investigation involved exhaustive taxonomic identifications and cell counting using the traditional microscopical method. Through the construction of 18S rDNA gene clone libraries for three selected samples along the surface salinity gradient, the genetic diversity of microeukaryotes was explored, providing a first insight into the protistan diversity that may occur in such a transition zone. The data offered an opportunity to compare the parallel use of morphological and molecular approaches for identifying taxa and measuring diversity in transitional waters.

## Materials and Methods

### 1. Ethics Statement

The present study was not carried out in a protected area or on private land. Therefore, no specific permission was required. We confirmed that the field study did not involve endangered or protected species. Only water samples were collected (no animals), therefore, not subject to regulation.

### 2. Study area and sampling strategy

**Figure 1:**
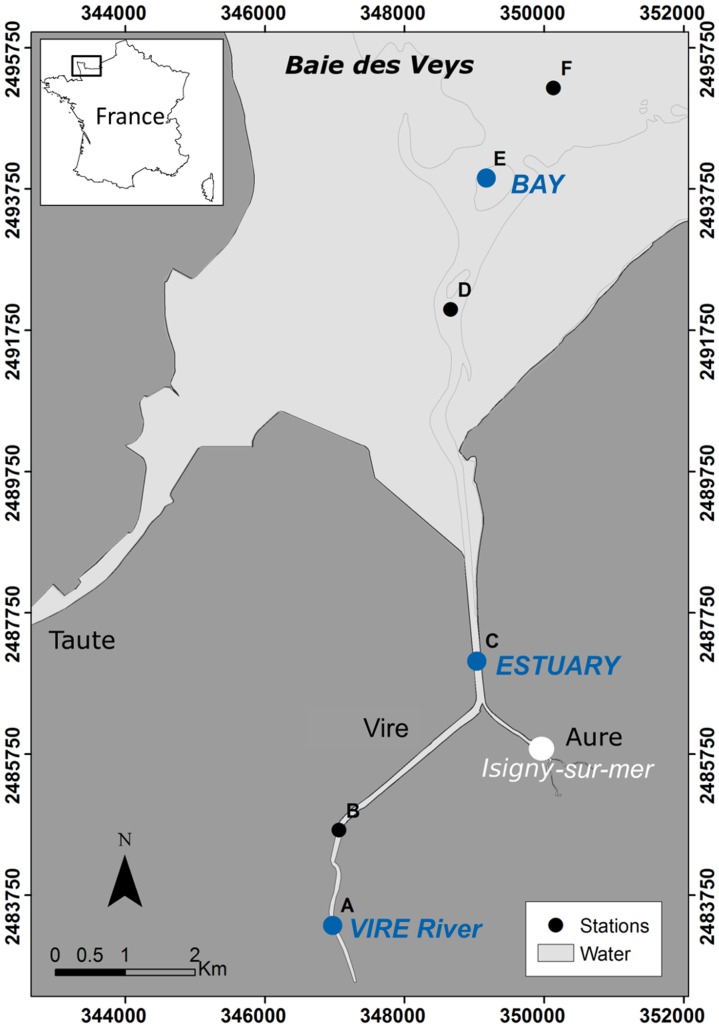
Map of the Vire River estuary (Baie des Veys) with location of the sampling stations (A–F). Blue dots indicate sampling points analyzed by both microscopy and clone library approaches (referred to as VIRE River, ESTUARY and BAY). Lambert II coordinates system.

The *Baie des Veys* is an intertidal estuarine ecosystem of the eastern English Channel, located in Normandy, north-western France (Figure 1). With a maximum tidal range of 8 m and a small intertidal area (37 km^2^, [20]), this macrotidal estuary is highly tide-influenced. Freshwater that enters the southern part of the bay derives from the discharge of four rivers, notably the main river, the Vire (length  = 128 km), which has an annual mean discharge of 15 m^3^ s^−1^, with important variations throughout the year. The freshwater inputs from this channel are the main source of nutrients in the bay and induce each year a relatively high primary production during the spring diatom bloom [18].

Sampling was undertaken on 27^th^ April 2010, at the end of the high river discharge period (4.85 m^−3^ s^−1^) and during the slack high tide, so that no tidal variation influenced our measurements. Six sampling sites (stations A–F, Figure 1) were established covering the whole salinity gradient of the Vire River estuary. Water was collected at the surface of all stations (samples As→Fs) and at 1 m above the bottom for stations B to E (samples Bb→Eb) with a 5-L Niskin bottle (stations A and F were only sampled at the surface due to technical constraints). All samples were analyzed by microscopy and three of the surface samples (As, Cs and Es) belonging respectively to the freshwater, brackish water and marine part of the estuarine gradient, were also used for clone library analysis (hereafter referred to as VIRE River, ESTUARY and BAY, Figure 1).

### 3. Physicochemical and biological measurements

Physicochemical profiles (depth, temperature, salinity) of the water column were obtained with a multi-parameter probe (Hydrolab Data sonde 5 Options, USA), and vertical profiles of irradiance were measured with an underwater quantum sensor (LICOR LI-1400, Nebraska, USA). Inorganic nutrients analysis was performed in the laboratory with a *Bran+Luebbe* Autoanalyzer AA3 according to Aminot & Kérouel [21]. Chlorophyll *a* biomass (Chl *a*) was estimated by fluorometry (Trilogy 7200-000 - Turner Designs, California, USA) according to the method of Welschmeyer [22] as described in Bazin et al. [23].

### 4. Morphological identification of the phytoplankton community

First observations with light microscopy (LM) on living material were carried out to establish a preliminary floristic list. For further identification and counting, sub-samples were rapidly fixed after collection with glutaraldehyde (final concentration 1%) and stored in darkness at 4°C until analysis. Phytoplankton was identified to the lowest possible taxonomic level using appropriate literature and keys for marine and freshwater environments (e.g. [24]–[26]). Further examination was made by scanning electron microscopy (SEM) using a JEOL JSM-6400. Heterotrophic dinoflagellates were included in the overall analysis.

Cells were enumerated using the Utermöhl settling method [27]. Because phytoplankton density can vary considerably along the estuarine gradient, volume of samples and settling time were adjusted (3 mL–10 mL for at least 48 hours) to ensure the complete sedimentation of the organisms [28]. Taxa were quantified at 400× in randomly-selected microscopic fields, with a Leica DMI3000B inverted microscope. A minimum of 500 individual units were counted, leading to a counting error not exceeding 10% [29].

### 5. DNA collection, PCR and cloning

To examine the overall eukaryotic community in the three selected surface samples (VIRE River, ESTUARY and BAY), 500 mL to 2 L of water was filtered onto 0.7-μm pore size glass fiber filters (Whatman) with no initial prefiltration.

As described in detail in Bazin et al. [23], total DNA was extracted using the Invisorb Spin Plant mini Kit (Invitek, Berlin, Germany) with modification of the first steps of the manufacturer's protocol, including cutting filters into pieces and cell disruption by thermal shocks (three freeze-thaw cycles: liquid nitrogen/+65°C).

Eukaryotic 18S rRNA genes (≈1800 bp) were amplified using the eukaryotic-specific primer set Euk A/Euk B [30] according to Bazin et al. [23]. Reactions were performed at two different annealing temperatures: 55 and 50°C, and pooled. The conditions were as follows: an initial hot-start at 95°C for 10 min, followed by 30 cycles (95°C for 1 min, 1.5 min at 50/55°C, 72°C for 2 min) and a final extension at 72°C for 10 min. Several replicates of PCR products were pooled and cleaned with the Wizard PCR clean-up system kit (Promega).

Clone libraries were constructed using the pCR2.1 TOPO-TA cloning kit (Invitrogen, Carlsbad, CA, USA) according to the manufacturer's instructions. The presence of inserts in the putative positive colonies was checked using flanking vector primers (M13). PCR products containing amplicons of the target size were purified and sequenced with an ABI Prism 3100 (Applied Biosystems, Foster City, CA, USA). The 18S rDNA was partially sequenced using the internal standard primer 895R (5′-AAATCCAAGAATTTCACCTC-3′) which covers conserved and rapidly evolving regions, and resulted in reads of 500–800 bp.

### 6. Taxonomic affiliation and phylogenetic analyses

All the sequences were manually checked, trimmed and edited using the SeqAssem software [31], and then compared to those available in public databases (GenBank) using the NCBI BLASTn web application [32]. Potential chimeras were detected with the online softwares Bellerophon [33] and KeyDNAtools [34]. After removal of low-quality sequences, metazoan sequences, and suspected chimeras, the remaining sequences were aligned using the slow and iterative refinement method FFT-NS-I with MAFFT 6.9 software [35]. The resulting alignment was checked and corrected manually. Based on this alignment, the sequences were clustered into distinct operational taxonomic units (OTUs) with MOTHUR 1.13 [36] using a similarity threshold of 98% that roughly corresponds to the genus/species level [37].

Phylogenetic trees including additional selected sequences from both GenBank and ARB databases were reconstructed using both neighbor joining (NJ, Jukes-Cantor distance) and maximum likelihood method (ML) with MEGA 5. Bootstrap support values (BP) were calculated from 1000 replicates for NJ tree and were reported on the ML tree, for which BP were from 100 replicates. The sequences reported in this paper have been deposited in the GenBank database under accession numbers JX645081- JX645156.

### 7. Diversity and statistical analyses

For the morphological approach, taxa richness and abundance were determined from floristic lists and cell counts. Taxa richness for the molecular data was estimated with MOTHUR, based on OTUs defined at 98% sequence similarity level. Rarefaction curves (Sobs) were generated for the three clone libraries and OTU richness was estimated by the non-parametric Chao 1 diversity estimator [38]. The comparison of taxa diversity between the samples was carried out through the Jaccard similarity index, by considering taxa (and OTUs) composition only. Principal Component Analysis (PCA) was performed to group samples according to the environmental variables (standardized and ln(x+1) transformed), and Correspondence Analysis (CA) was applied to a matrix of the relative contribution of phytoplankton in order to establish relationships between taxonomic composition and sampling stations. All statistics were computed with the PAST software [39].

## Results

### 1. Environmental context

**Figure 2:**
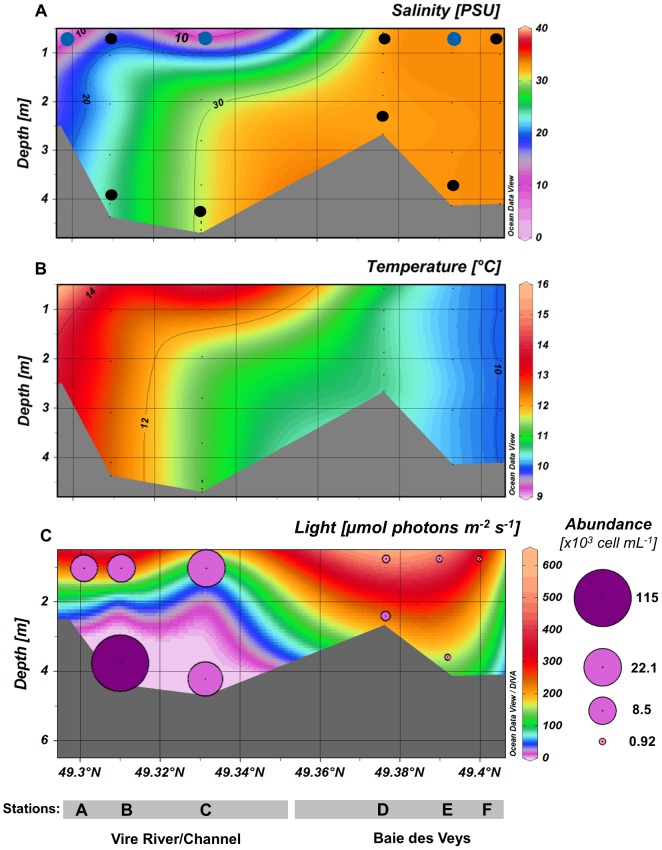
Profiles of salinity (A), temperature (B), irradiance and phytoplankton abundance (C) along the estuarine gradient. (Stations A→F). Black dots on the profile (**A**) represent sampling points collected for the global phytoplankton analysis by microscopy, and the blue dots indicate those analyzed by both microscopy and clone library approaches. A logarithmic scale was used for the representation of phytoplankton cell abundance on the profile of irradiance(**C**).

The Vire River estuary is characterized by shallow waters (depth  = 2.7–4.7 m along the whole transect at the time of sampling) (Figure 2). Clear horizontal and vertical gradients characterized the distribution of salinity, temperature and nutrients (Figure 2A–B and Figure S1) in the first part of the transect (stations A–D), emphasizing the water column stratification (i.e. a two-layer system) within the Vire channel at high tide. As confirmed by the PCA analysis including nine environmental variables (temperature, salinity, nitrate, nitrite, phosphate, silicate, Chl *a* concentrations and total phytoplankton abundance, Figure S2), three main area can be discriminated along the estuarine continuum. An “upper surface layer” in the river channel [samples As( = VIRE River), Bs, Cs( = ESTUARY)], defined by oligohaline (0.5–5) to mesohaline (5–18) waters, high nutrients from terrestrial runoff, warmer temperatures (13.8–16°C) and high phytoplankton abundance (8.5–22×10^3^ cells mL^−1^), was distinguished from the bottom brackish layer (samples Bb-Cb), which was characteristic of the estuarine “silt plug” (or estuarine turbidity maximum, ETM). The latter was characterized by polyhaline water (18–30) and total darkness due to very high light attenuation in the water column (attenuation coefficient Kd  = 2.4–1.8 for B–C, knowing that Kd  = 0.035 for pure water) associated with highest phytoplankton abundance (>115×103 cells mL^−1^ in Bb) (Figure 2C). No vertical gradient was noted for stations D–F (including sample Es  =  BAY) due to well-mixed water column within the bay (Figures 2, S1 and S2). This “coastal” area was defined by higher salinities (euhaline >30) but lower temperatures (<11°C), as well as lower nutrients, Chl *a,* and phytoplankton abundances (≤1.2×10^3^ cells mL^−1^).

### 2. Spatial pattern in phytoplankton community structure

**Figure 3:**
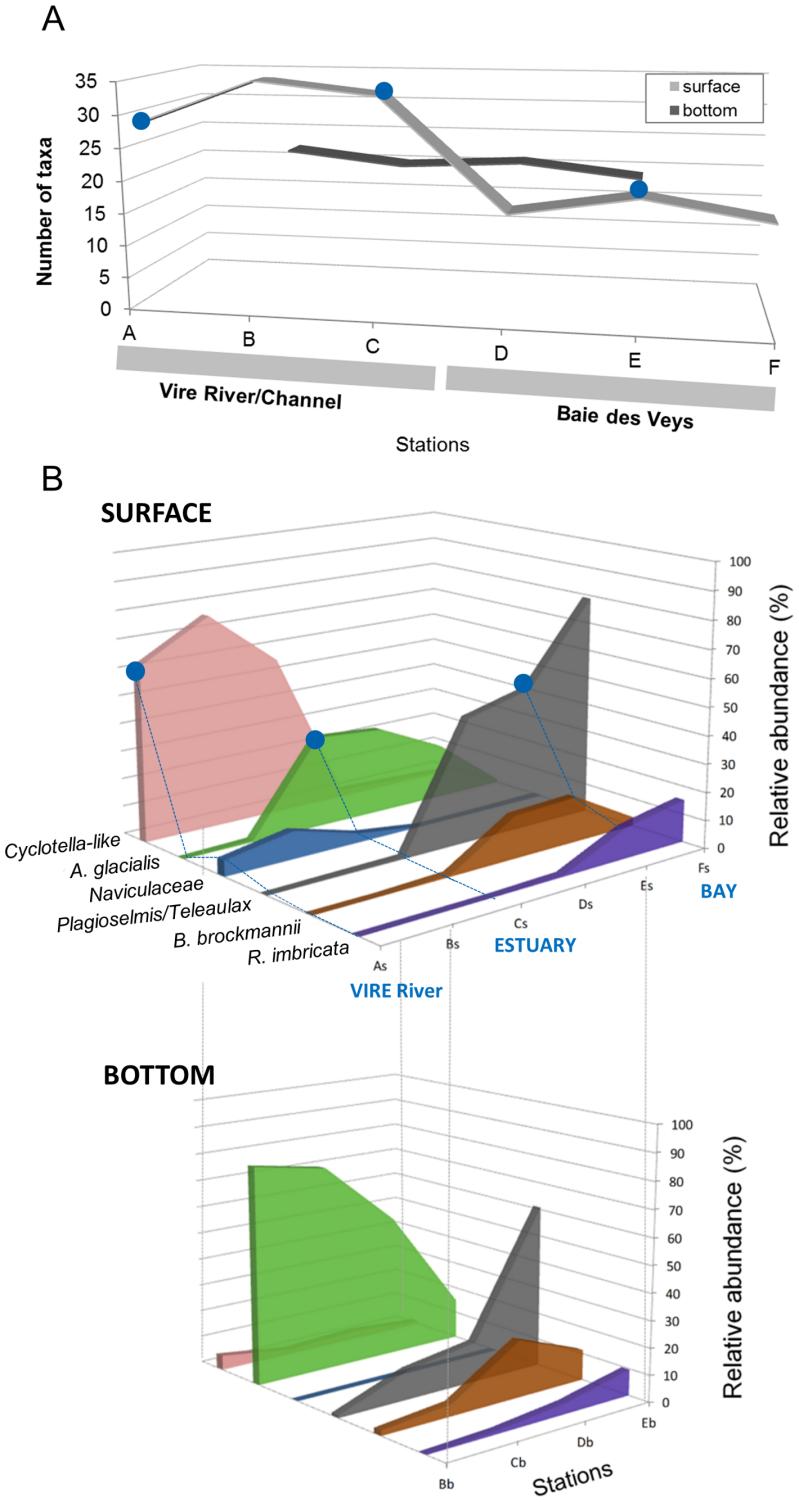
Taxa richness and distribution of dominant species along the estuarine gradient. (A) Total phytoplankton richness. (B) Relative abundances of dominant taxa (accounting for >10% of total phytoplankton in at least one sample) at the surface (top) and depth (down). *A. glacialis*  =  *Asterionellopsis glacialis*, *B.brockmanni*  =  *Brockmanniella brockmannii* and *R.imbricata*  =  *Rhizosolenia imbricata.* Blue dots indicate sampling points analyzed by both microscopy and clone library approaches (referred to as VIRE River, ESTUARY and BAY).

A distinct horizontal gradient in algal cell abundance (Figure 2C), taxa richness (Figure 3A) and taxa composition (Figure 3B) was observed along the whole transect, with vertical variations in the water-stratified zone in the Vire channel (B–C). A total of 86 different species were observed (floristic list, Table S1) corresponding to 63 genera, and six taxa were regarded as dominant species (>10% in at least one sampling point, Figure 3B).

#### Major taxa in the marine section

Lowest taxa richness and abundances (<1.2×10^3^ cells mL^−1^) were found in the Baie des Veys (stations D–F, including BAY) (Figures 2C, 3A). The Cryptophyceae, exclusively represented by the *Teleaulax/Plagioselmis* group, dominated the phytoplankton community in the northern part of the bay reaching 79% of total species abundance in Fs (Figure 3B). Predominance of these flagellates was reduced toward the mouth of the estuary, where a shift towards dominance of diatoms (Bacillariophyceae) was observed with a relative contribution reaching 84% at the bottom of station D. The most abundant diatoms in the bay were the pennates *Asterionellopsis glacialis* (max.  = 53% of total species in Db), *Brockmanniella brockmannii* (21% in Db) and the centric *Rhizosolenia imbricata* (15% at Fs) (Figure 3B).

Due to similarity in species composition, samples from the north of the bay (E–F), regardless of the depth, were grouped in the same cluster on the CA ordination diagram (Figure S3). This highlights the well-mixed water column in the coastal part of the transect unlike the stratified mixing zone in the channel.

#### Major taxa in the brackish stratified section

Bacillariophyceae were the major class in the mixing zone (stations B–C), but clear differences in taxa composition were detected between surface and depth (Figures 3 and S3).

In the brackish bottom water (Bb-Cb) derived from the bay and moving landward in the Vire River channel, cell abundances increased sharply (Figure 2C). This formed a dramatic phytoplankton accumulation in the bottom water of station B (silt plug) predominated by *Asterionellopsis glacialis* (76%–81% of the total phytoplankton, Figure 3B) with more than 115×10^3^ cells mL^−1^, i.e. 100 to 200-fold higher than in the bay.

The surface brackish layer Bs-Cs (ESTUARY), influenced by the upstream river, showed the highest phytoplankton diversity observed along the transect (Figure 3A), reaching twice as many species than in the north of the bay (Bs vs. Fs). A large part of this diversity (up to 69%) comprised taxa occurring at low cell concentrations ( =  rare taxa, <1% of total species abundance). The genus *Cyclotella* and associated small centric diatoms (designated here as *Cyclotella*-like) were predominant (57–76%, Figure 3B). Further SEM observations of this group led to the identification of a great diversity of morphologically similar small taxa (<10 μm for the most part), not identifiable under the light microscope, e.g.*: Cyclotella meneghiniana, Discostella* sp., *Cyclostephanos dubius, Stephanodiscus minutulus, S. hantzschii, Thalassiosira guillardii.* Presence of green algae from the class Chlorophyceae was significant (3–7.5% of total abundance), including the genera *Monoraphidium*, *Scenedesmus* and *Chlamydomonas*.

According to the CA ordination (Figure S3), Cs (ESTUARY) had an intermediate place on the diagram with no typically occurring species, while Bs plotted close to the freshwater sample As, with chlorophyte species and freshwater diatoms as most typical taxa.

#### Major taxa in the freshwater section

The *Cyclotella*-like group was predominant in the freshwater station A =  VIRE River (63% of total abundance). Other noteworthy diatoms were the freshwater species *Nitzschia acicularis* (7%) and some unidentified Naviculaceae (6%). Compared to the other stations, chlorophytes were most abundant (20.8% of total abundance), including members of the Chlorophyceae (12%) and Trebouxiophyceae (9%).

#### Other algal groups

The other taxa identified, including dinoflagellates (4 taxa), haptophytes (2), chrysophytes (2), and euglenophytes (1), were much less abundant along the transect (<1% of total abundance).

### 3. Clone libraries analysis

The following three eukaryotic rDNA clone libraries were constructed: the “**VIRE River”** library from the upstream freshwater sample As (1 psu), **“ESTUARY”** from the brackish sample Cs (8.5 psu), and “**BAY”** from the marine sample Es (33 psu). After removal of low-quality results, a total of 287 partial microeukaryotic sequences were obtained. Clustering of sequences based on a 98% similarity level revealed a total of 67 different operational taxonomic units (OTUs, Table 1), with 37, 13 and 26 OTUs for VIRE River, ESTUARY and BAY respectively. Rarefaction curves computed for each library (to obtain an estimate of phylotype diversity relative to sampling effort, i.e. number of clones sequenced) never reached saturation (Figure S4). However, a comparison between the curves showed that higher microeukaryotic diversity was found in the freshwater and marine samples rather than in the brackish one. The non-parametric richness estimator Chao1 yielded values of 60, 27 and 61 OTUs for VIRE River, ESTUARY and BAY respectively. Therefore, the recovered OTUs represented only 43 to 62% of the estimated OTU richness (Chao1), meaning that an important part of the genetic protistan diversity remains unsampled. The ESTUARY library was more substantially undersampled because of the strong predominance of an OTU affiliated to *Thalassiosira guillardii,* which contained 68 sequences out of the 101 obtained.

**Table 1:** OTUs recovered in this study (clustering at a 98% similarity threshold).

									Distribution of clones			
OTU	Division	Class/or other group	Closest match	Accession	Identity (%)	Origin (for uncultured clones)	Closest cultured match	Identity (%)	Total	VIRE	ESTUARY	BAY
V99	Stramenopiles	Chrysophyceae	*Mallomonas portae-ferreae*	GU935618	99.6				1	1	0	0
B83			Uncult. eukaryote clone LG47-07	AY919816	99.8	Lake George, oligotrophic (USA)	*Paraphysomonas foraminifera*	99.4	4	1	0	3
V56			Uncult. eukaryote clone AMT15_15_10m	GQ863816	98.8	Upwelling region, Atlantic (Canaries)	*Paraphysomonas butcheri*	98.7	1	1	0	0
V09			Uncult. stramenopile clone MLB53.161	EU143922	97.4	Lake Taihu, subtropical (China)	*Uroglena* sp.	94.4	1	1	0	0
V92			Uncult. eukaryote clone RW2_2011	AB721074	96.5	Freshwater, Kiryu (Japan)	*Uroglena* sp.	96.1	2	2	0	0
V69			*Spumella*-like flagellate (isolate JBAS36)	AY651079	99.6	Freshwater, Nag Pokhari (Nepal)	*Pedospumella encystans*	99	2	2	0	0
V78			*Spumella*-like flagellate (isolate JBC27)	AY651093	93.3	Small pond, Huqiu (China)	*Chrysosphaera parvula*	90.9	2	2	0	0
V97			Uncult. eukaryote clone PG5.22	AY642735	97.5	Lake Godivelle, oligotrophic (France)	*Paraphysomonas imperforata*	95.1	1	1	0	0
V145			Uncult. eukaryote clone Ch8A2mF4	JF730784	99.2	Char Lake (Arctic)	*Ochromonas tuberculata*	95.5	13	13	0	0
V81			Uncult. eukaryote clone WH8eA1	JF730838	99.8	Ward hunt Lake (Arctic)	*Ochromonas tuberculata*	96.4	1	1	0	0
B18			Uncult eukaryote clone CYSGM-7	AB275090	98	Marine sediment, methane seep (Japan)	*Kephyrion* sp.	96.7	21	0	1	20
B59			Uncult. eukaryote clone Q2B03N10	EF172974	97.6	Sargasso Sea (25m depth)	*Synura curtispina*	91	1	0	0	1
Es64		Bacillariophyceae	*Thalassiosira guillardii*	AF374478	99.7				68	0	68	0
Es37			*Thalassiosira pseudonana*	DQ093367	99.8				4	0	4	0
V141			*Cyclotella meneghinia*	AY496207	100				6	6	0	0
V105			*Stephanodiscus hantzschii*	DQ514914	99.8				26	10	16	0
V38			*Discostella pseudostelligera*	DQ514905	99.8				10	7	3	0
Es86			*Thalassiosira nordenskioeldii*	DQ514886	99.9				1	0	1	0
Es109			*Diatoma tenuis var. elongatus*	EF423403	99.9				1	0	1	0
Es123			*Asterionellopsis glacialis*	X77701	99.8				1	0	1	0
Es110			*Navicula phyllepta*	EU938308	100				1	0	1	0
V154			*Navicula gregaria*	HM805037	99.9				1	1	0	0
V163			*Surirella brebissoni*	AJ867029	99.7				1	1	0	0
B128			*Rhizosolenia imbricata*	AY485510	100				7	0	0	7
B140		Bolidophyceae	Uncult. eukaryote clone BLACKSEA_48	HM749950	99.5	Southern Black Sea	*Bolidomonas pacifica*	95.7	1	0	0	1
V16		Bicosoecids	Uncult. eukaryote clone P34.6	AY642710	98.1	Lake Pavin, oligomesotrophic (France)	*Adriamonas peritocrescens*	93.4	1	1	0	0
V44		MAST-2	Uncult. eukaryote clone W8eD9	JF730854	99.9	Ward hunt Lake (Arctic)	*Pirsonia verrucosa*	88.5	1	1	0	0
B110		MAST-4	Uncult. eukaryote clone H3S8Ae5	JQ781882	100	North pacific ocean	*Pirsonia verrucosa*	88.9	1	0	0	1
B75		MAST-12	Uncult. eukaryote clone BAQA21	AF372755	96	Marine anoxic sediments, Berkeley (USA)	*Oblongichytrium* sp.	84.1	1	0	0	1
V122	Cryptophyta	(nucleus)	*Cryptomonas curvata*	AM051189	99.9				21	21	0	0
V83			*Cryptomonas pyrenoidifera*/*C. marssonii*	EU163587/AM051192	99.7				1	1	0	0
V42			*Cryptomonas tetrapyrenoidosa*/*C. ovata*	AB240954/AF508270	100				3	3	0	0
V151			*Cryptomonas borealis*/*C. ovata*	AM051188/AB240952	93.9				1	1	0	0
V23			Uncult. Cryptophyte, clone STFeb_146	HM135081	99.9	Lake Stechlin, oligotrophic (Germany)	*Teleaulax amphioxeia*	98.7	1	1	0	0
V113			Uncult. eukaryote, clone D7	FN263278	100	Southern Baltic sea	*Geminigera cryophila*	99.6	6	1	1	4
B67			*Teleaulax acuta*	AB471786	100				3	0	0	3
B147			Cryptophyceae sp.	GQ375265	99.7	Northern Baffin Bay (Arctic ocean)	*Falcomonas daucoides*	98.6	3	0	0	3
B96		(nucleomorph)	*Falcomonas daucoides*	AJ420689	86.2				2	0	0	2
B23	Haptophyta	Prymnesiophyceae	*Haptolina hirta*	AJ246272	98.7				1	0	0	1
B121			*Phaeocystis globosa*	AF182112	99.6				1	0	0	1
B01	Picobiliphytes		Uncult. eukaryote clone RA000907	DQ222877	99.2	English Channel			1	0	0	1
B107	Centrohelids		*Pterocystis* sp. unidentified helio 5	AY749610	95.6	Okareka Lake (New Zealand)	*Heterophrys myriopoda*	94.9	1	0	0	1
V33	Alveolata	Dinophyceae	*Gymnodinium eucyaneum*	JQ639760	99.8				1	1	0	0
B131			*Gyrodinium spirale*	AB120001	99.7				8	0	0	8
B49			Uncult. eukaryote clone M1_18B06	DQ103837	99.5	The anoxic Mariager Fjord (Denmark)	*Peridinium umbonatum*	93.4	5	0	0	5
B115			Uncult eukaryote clone SA1_4B9	EF527151	99.4	The anoxic Framvaren Fjord (Norway)	*Euduboscquella* sp.	96.2	1	0	0	1
V98		Ciliophora	Uncult. eukaryote clone VNP11	DQ409125	99.4	Lacustrine reservoir, hyper-eutrophic (France)	*Strobilidium caudatum*	90.1	2	2	0	0
V84			Uncult. alveolate clone 1-D5	FN689891	99.8	Gulf of Finland (Finland)	*Tintinnidium* sp. 1	90.5	1	1	0	0
V85			Uncult. ciliate clone AY2009D10	HQ219435	100	Lake Aydat, eutrophic (France)	*Strobilidium caudatum*	90	1	1	0	0
V61			Uncult. eukaryote clone VNP38	DQ409135	100	Lacustrine reservoir, hyper-eutrophic (France)	*Pseudochilodonopsis fluviatilis*	93.2	2	2	0	0
B116			*Tintinnopsis rapa* (isolate 241)	JN831834	99.4				2	0	0	2
V127	Chlorophyta	Chlorophyceae	*Chlamydomonas noctigama*	JN903979	99.7				2	2	0	0
V102			Uncult. eukaryote clone KRL01E11	JN090871	100	Lake Karla (Greece)	*Chlamydomonas kuwadae*	99.4	7	5	1	1
V140			*Neochlorosarcina negevensis*	AB218715	99.1				1	1	0	0
V146			*Mychonastes huancayensi*/*M. jurisii*	GQ477050/GQ477038	99.9				2	2	0	0
V131			*Atractomorpha echinata*	AF302772	100				1	1	0	0
Es74		Prasinophyceae	*Nephroselmis olivacea*	X74754	98,1				1	0	1	0
B125			Uncult Crustomastix, clone PROSOPE.CM-5m.	EU143398	96.9	Mediterranean Sea	*Crustomastix stigmatica* (pras1)	94.6	1	0	0	1
B129			*Micromonas pusilla strain*	AY955010	99.7				1	0	0	1
V05	Fungi	Chytridiomycota	Uncult. eukaryote clone B86-172	EF196796	98.2	Freshwater, Alpen (France)	*Spizellomyces pseudodichotomus*	94.4	1	1	0	0
Es32			Uncult. Chytridiomycota clone PFD5SP2005	EU162640	99.6	Lake Pavin, oligomesotrophic (France)	*Kappamyces laurelensi*	94.8	9	7	2	0
V31		Environmental clade LKM	Uncult. eukaryote clone E-C4_1	HM628660	99.7	Slow sand filter biofilm	*Mortierella microzygospora*	87.1	2	2	0	0
V136		Unknown lineage	Uncult. fungus clone C10	JN054676	100	Wastewater treatment plant	*Rhizophlyctis rosea*	87.5	1	1	0	0
B64	Cercozoa	Phytomyxea	Uncult. eukaryote clone TAGIRI-5	AB191413	96.3	Anoxic marine sediment (Japan)	*Phagomyxa bellerocheae*	87.8	1	0	0	1
B65		Filosoa	Uncult. eukaryote clone 9_149	EU545751	99.8	Marine sediment, East Sea	*Botuliforma benthica*	95,4	1	0	0	1
B87			Uncult. eukaryote clone 9_25	EU087251	100	Marine sediment, East Sea	*Protaspis oviformis*	98,8	3	0	0	3
B144			*Thaumatomastix* sp.	GQ144681	100	Boundary Bay (Canada)	*Thaumatomonas coloniensi*	94.4	1	0	0	1

A representative clone for each OTU and its phylogenetic affiliation are provided. Highest BLAST match and closest cultured organisms from GenBank are given with accession numbers and percentage similarity. The remaining columns indicate the distribution and the number of clones found in each clone library (VIRE River, ESTUARY, and BAY).

Among the 287 sequences identified, up to 94% (falling in 56 OTUs) had at least 98% similarity with known sequences based on BLAST analysis, and were widely distributed across the major eukaryotic lineages. The following first-rank taxa were represented: stramenopiles, Cryptophyceae, Haptophyta, Alveolata, Chlorophyta, Fungi, Cercozoa and two subgroups of eukaryote *incertae sedis* (centrohelids and picobiliphytes) (Table 1). However, 30 out of the 68 total OTUs had less than 98% similarity with named and cultured organisms, meaning that sizeable fractions of protistan groups in the environment still remain to be sequenced and characterized. Most of these clones belonged to heterotrophic representatives (e.g. Fungi, Ciliates, Cercozoa, some Dinophyceae and stramenopiles). Overall, the heterotrophic/mixotrophic OTUs accounted for 44% of all OTUs.

Total sequences were dominated by stramenopiles [accounting for 182 clones (64%) falling in 29 OTUs (43%)], followed by alveolates (9% of all clones in 9 OTUs), cryptophytes (14% of all clones in 8 OTUs) and chlorophytes (<6% of all clones in 8 OTUs). The other taxonomic groups were less abundant in the libraries, each accounting for less than 5 OTUs.

Stramenopiles. The 29 stramenopiles OTUs retrieved from our study were distributed across four major lineages (Table 1). The photosynthetic groups included the diatoms (Bacillariophyceae, 12 OTUs) detected in all libraries but predominant in the estuarine sample (74% of all diatom sequences, 8 OTUs), and the picoplanktonic class Bolidophyceae as a singleton (i.e. an OTU containing only one sequence) in the bay. All the diatom sequences showed high similarity with cultivated strains (99.7–100%) and were for the most part affiliated with genera also identified by microscopy in this study. Half of the diatom OTUs (6) belonged to thalassiosiroid lineages found in the Vire channel (3 OTUs in ESTUARY, 1 OTU in VIRE River, and two present in both libraries), including several Stephanodiscaceae (*Cyclotella meneghiniana*, *Discostella pseudostelligera*, *Stephanodiscus hantzschii*) and small representatives of Thalassiosiraceae (e.g. *T. pseudonana*, *T. guillardii*). *Thalassiosira guillardii* was the predominant OTU in ESTUARY (67% of total clones) but not detected in the other libraries. Only one diatom OTU was found in BAY and belonged to *Rhizosolenia imbricata,* which was the most abundant diatom species detected by microscopy in this part of the bay.

Non-photosynthetic groups of stramenopiles were represented by singletons belonging to Bicosoecida and three lineages of uncultured novel Marine Stramenopiles (MASTs). MAST-2 is a clade hitherto known from exclusively marine waters, found in diverse oceanic and coastal areas [40], [41]. However, our analysis showed that the clone V44 from VIRE River, together with two other freshwater clones (99.9% similarity) recovered from an Arctic lake (W8eD9, [42]) and an oligotrophic temperate lake (STFeb_251, [43]), clustered with high bootstrap values with marine MAST-2 sequences (Figure S5). Clone B75 from the bay was positioned within the radiation of MAST-12, which is mainly composed of sequences from oxygen-depleted habitats [44], and the clone B110 belonged to the MAST-4 lineage known to be widely distributed in temperate samples [45].

**Figure 4:**
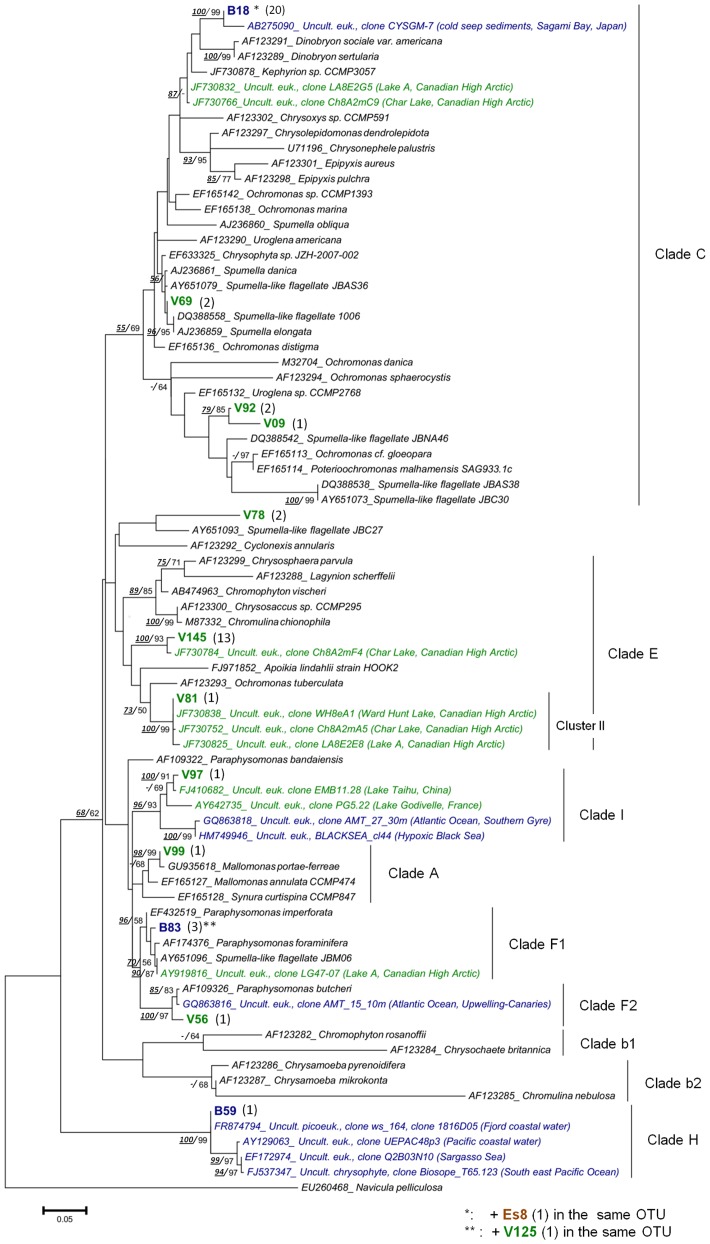
Maximum likelihood (ML) tree showing the position of the Chrysophyte OTUs. OTUs were obtained from the VIRE River (**V**), the ESTUARY (**Es**) and the BAY (**B**) clone libraries. Tree construction was based on an alignment of 79 partial sequences (ca 500 align positions). The diatom *Navicula pelliculosa* was used as outgroup. The number of clones per OTU is indicated in brackets. Sequences from cultured taxa appear in black and environmental sequences in green (freshwater), blue (marine) or brown (brackish/estuary). Bootstrap values (>50%) obtained from the neighbor-joining tree and those from ML tree are indicated (***NJ*** **/**ML).

The chrysophytes *sensu lato* (i.e. including Synurophyceae) were represented by 50 clones falling in 12 OTUs, and were therefore, with the diatoms, the most diverse lineage detected in this molecular survey (Table 1). Most of these OTUs (10) were recovered from VIRE River, while 3 OTUs were detected in BAY, and only one in ESTUARY. Phylogenetic analyses placed these sequences in seven clades among the twelve recently proposed by del Campo & Massana [46] (Figure 4), with a tree topology in agreement with those previously described [46], [47]. An OTU from the freshwater library (V99) belonged to the clade A ( =  Synurophyceae) containing photosynthetic organisms, and was closely related to *Mallomonas portae-ferrae* (99.6% similarity). Two OTUs (V56, B83) were closely related to cultured organisms of the heterotrophic genus *Paraphysomonas* (clades F1, F2), while four others OTUs were affiliated to clade C, but their internal position within the clade remains uncertain. Among the latter, clone B18 was representative of the predominant OTU (20 clones) in BAY library, which was moderately related to loricate organisms such as *Kephyrion* sp. [42] (96.7% similarity), and the riverine clone V69 was highly similar to non-green ‘*Spumella-*like’ strains, all isolated from soil (99.6% similarity with JBAS36 [48]). Three OTUs (V81, V97, and B59) belonged with high bootstrap support (96–100%) to three lineages (cluster II, clade I, clade H) containing only environmental sequences to which they were closely related (closest matches: 97.5–99.8%). Among them, the freshwater OTU V81 fell into the cluster II containing sequences solely recovered from high Arctic lakes [42]. The clone V97 together with other sequences from freshwater systems (subtropical or oligotrophic lakes) clustered with high support with marine sequences (Black Sea, Atlantic Ocean) previously described as members of the clade I [46] or ‘Marine B’ [49]. This clade was until now defined as exclusively marine; however, the present phylogenetic analysis showed that it actually contains both freshwater and marine representatives.

#### Alveolates

Nine OTUs were affiliated to alveolates but no sequences were found in the ESTUARY library (Table 1). Dinophyceae were represented by 4 OTUs, among which two were closely related to the cultivated and known species *Gymnodinium eucyaneum, Gyrodinium spirale*. The two other dinoflagellate OTUs (B49, B115, Table 1) showed rather high similarities with uncultured clones from anoxic fjord waters [10], [50]. Among them, B115 was related to the syndinean *Euduboscquella* sp known to be a parasite of tintinnids [51].

#### Cryptophyceae

**Figure 5:**
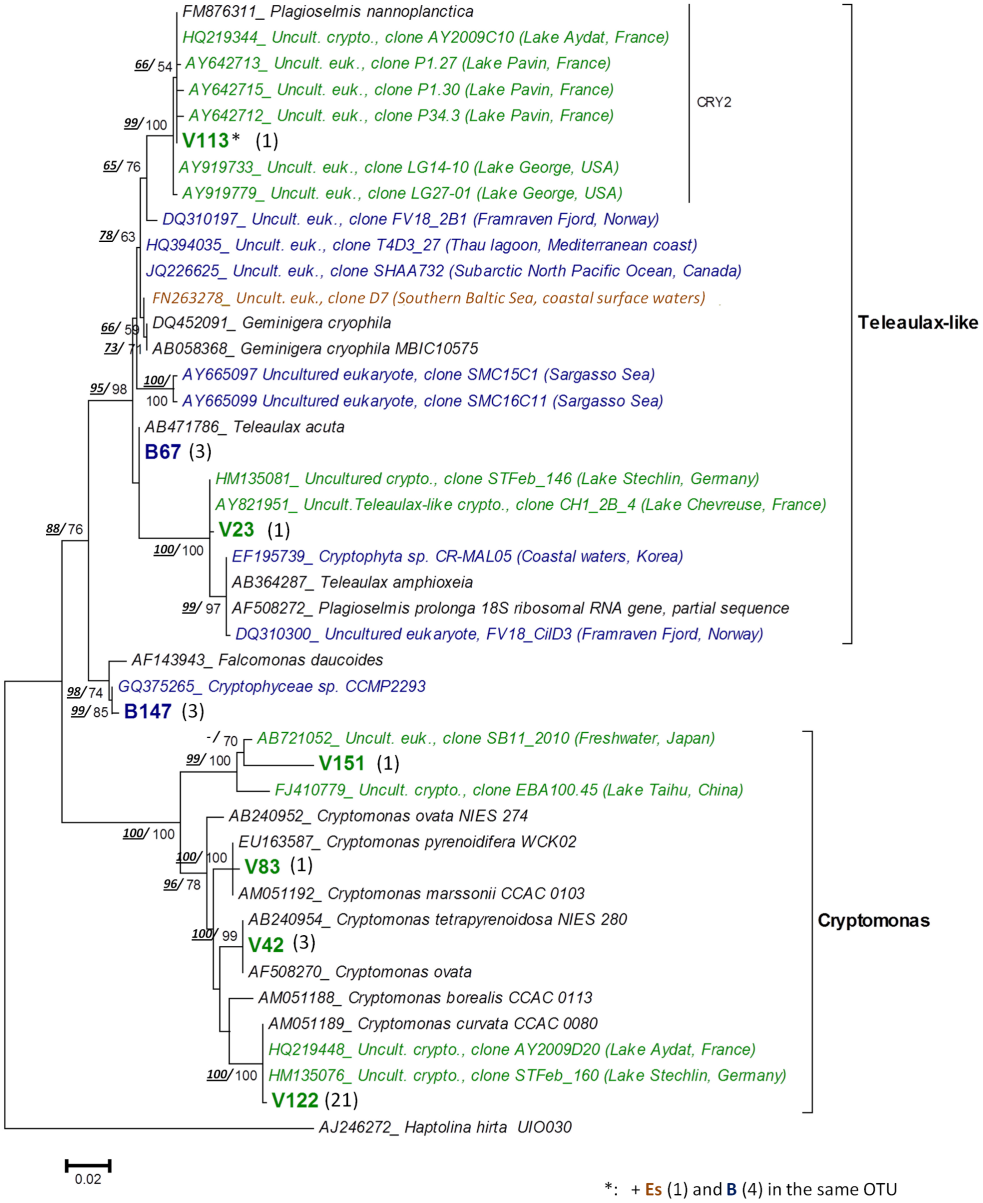
Maximum likelihood (ML) tree showing the position of the Cryptophyte OTUs (order Cryptomonadales). Tree construction was based on an alignment of 44 partial sequences (ca 650 align positions). The haptophyte *Haptolina hirta* was used as out-group. (See legend of Figure 4 for details).

A total of 8 OTUs were affiliated with nuclear or nucleomorph cryptophyte sequences, and distributed in three major lineages within the order Cryptomonadales (Figure 5, Table 1). Four OTUs recovered from the Vire River were closely related to *Cryptomonas* species, e.g. *C. ovata* and *C. curvata;* (99.7–100% similarity) and rather moderately *C. borealis* (93.9% similarity). An OTU from the river (V23) along with one unique to BAY library (B147) and another present in all clone libraries (representative: V113), belonged to the *Teleaulax*-like cluster (bootstrap support 98%, Figure 5) that includes *Geminigera* and *Plagioselmis* genera lineages [52]. Another OTU from the bay (B147) was closely related to the species *Falcomonas daucoides* (98.6%).

#### Chlorophyta

Most of the chlorophyte sequences (Table 1) were retrieved from the VIRE River library (11 clones) and were affiliated to the class Chlorophyceae, falling in 5 OTUs with high similarity to known genera (from 99 to 100%). They corresponded either to small sized coccoid species such as *Mychonastes* sp. and *Neochlorosarcina negevensis,* or to the flagellated *Chlamydomonas* species. The class Prasinophyceae was detected as singletons in the brackish and marine libraries, including clones highly similar to *Micromonas pusilla*, *Nephroselmis oliveacea,* and to an environmental clone of the *Crustomastix* lineage [34].

#### Other groups

Among the remaining OTUs, two singletons from the BAY library were affiliated to the haptophytes (Table 1), both related to members of the Prymnesiophyceae (*Phaeocystis globosa, Haptolina* sp.). Finally, the marine picoplanktonic group Picobiliphytes was represented by an OTU in the bay (B01), highly similar to an environmental clone (99.2% similarity with RA000907.54) recovered from the English Channel [53].

### 4. Molecular versus Morphological diversity

**Figure 6:**
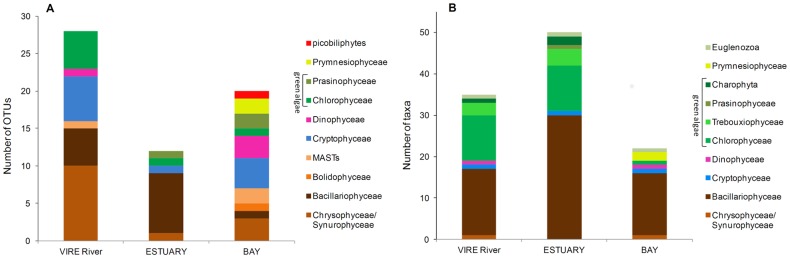
Phytoplankton taxonomic composition (taxa richness) in three surface samples: Molecular vs. Morphological approaches. VIRE River (station A), ESTUARY (station C) and BAY (station E). (A) Number of OTUs according to the taxonomic affiliation of the 18S rRNA gene sequences. (B) Number of taxa identified by microscopy (LM + SEM).

To compare diversity estimates by microscopical and molecular methods, sequences from exclusively heterotrophic lineages were removed from analysis (e.g. Fungi, Cercozoa, and Ciliates). Taxa richness estimated with both approaches showed substantially different trends between the three samples (Figure 6). Microscopic analyses (LM+SEM) revealed a much higher taxonomic richness in the ESTUARY sample, whereas, conversely, number of OTUs found in ESTUARY was lower than in both VIRE River and BAY libraries.

Taxa composition of the different communities at a high taxonomy level (class/division, Figure 6) showed differences between the two approaches. The clone library analysis identified a greater diversity within stramenopiles, including lineages of nano- and/or pico-size flagellates (Chrysophyceae, MASTs, Bolidophyceae). Moreover, in spite of being identified by microscopy, a greater diversity within Cryptophyceae and Dinophyceae was also recovered with the molecular survey. On the other hand, diatoms and green algae were better detected with the morphological approach.

Only three OTUs were shared between the clone libraries VIRE River and ESTUARY (Table 1), and one between ESTUARY and BAY, reflecting a low similarity between communities (Jaccard index: 11% and 10% respectively). The microscopic survey indicated that 21 taxa were shared between VIRE River and ESTUARY (floristic list, Table S1) with a Jaccard's similarity of 39%, and 8 taxa were found in both samples ESTUARY/BAY resulting in a 18% similarity. The morphological survey therefore revealed higher similarity between both sample combinations than the 18S rDNA clone library approach did.

## Discussion

Our taxonomic investigation along the Vire River estuary and in the Baie des Veys took place just at the beginning of the diatom spring bloom, which usually occurs every year in the bay [18]. Physicochemical parameters and chl *a* concentrations estimated within the bay were consistent with previous studies carried out in the same season (for more details: [17]–[19]). Although we studied the estuarine continuum at a single date, by using two contrasting but complementing methods we enhanced our assessment of the global microeukaryote diversity in this site hitherto considered a relatively well-known ecosystem.

### 1. Community structure and diversity along the macrotidal Vire estuarine gradient in early spring bloom

Examination of species assemblages (see Figures 3 and S3) suggested a gradual change of the phytoplankton composition along the estuarine continuum influenced by salinity and tidal force. The latter involves a stratification of the water column at high tide, with significant vertical differences both in hydrographic and biological data. This two-layer circulation system characteristic of this macrotidal estuary has been previously introduced byJouenne et al. [18]. At the time of sampling, water stratification within the mixing zone of the channel (stations B–C) was particularly pronounced because the Vire River discharge was low (<½ the mean annual discharge) compared to the tidal current (spring tide). Salinity thus increased rapidly in the freshwater surface layer, while seawater intrusion into the bottom layer extended upstream over 7 km from the mouth section (Figure 2). A spectacular phytoplankton bloom (>100×10^3^ cells mL^−1^) occurred in the most upstream brackish reach of the bottom seawater (station B bottom – 24 psu), with cell abundances among the highest reported in estuaries [54], [55]. This bottom zone of the estuarine gradient corresponds to the estuarine turbidity maximum (ETM) or “silt plug”, characterized by high organic matter accumulation resulting in strong light-limitation [15]. Presence of an ETM is reported here for the first time in the Vire River estuary.

During our study, three main communities and their associated water mass overlapped each other, longitudinally and vertically along the estuarine continuum.

Within the “coastal” community in the well-mixed water column of the Baie des Veys, diatoms were one of the dominant groups, as commonly found throughout the year [18]. However, we report here for the first time that the nanoplanktonic group *Teleaulax*/*Plagioselmis-*like (Cryptophyceae) was preponderant in this area. According to the clone library content, two different OTUs belonging to the *Teleaulax*-like cluster probably correspond to the taxa enumerated, including one closely related to *Teleaulax acuta*. Other sequences however corresponded to *Falcomonas daucoides*, a species with cells superficially resembling *Plagioselmis* and *Telelaulax* when observed by light microscopy, due to a similar acute posterior end [52]. Given this similarity, individuals of *F. daucoides* were probably included in the cell count of the *Teleaulax*-like group, although morphological differences could be revealed with electron microscopy [56]. This illustrates the limits of the quantitative approach with light microscopy that may overlook an important part of the overall diversity [43], [57]. It also reflects the need to further investigate this cryptophyte group, whose importance and recurrence as part of marine/estuarine communities has been recently emphasized (reports of the dominance of these flagellates is increasing[58]), but remains largely ignored due to difficulties in taxonomic identifications and suspicions of several phylogenetic misclassifications [59].

Shift in species composition occurred toward the river mouth and the channel, where the two-layer system in the mixing zone led to the presence of two different phytoplankton communities.

A “bottom-brackish” community was mainly composed of coastal euryhaline species transported at high tide by the tidal current into the bottom of the Vire channel.

The unexpected phytoplankton bloom restricted to the polyhaline bottom layer (24 psu) in the ETM zone was mainly composed of the euryhaline diatom *Asterionellopsis glacialis* (81% of the total phytoplankton), while typical marine species disappeared as they were advected upstream. Low irradiance caused by high turbidity in the silt plug can constrain development of phytoplankton and prevent it from using available nutrients [60], however, our microscopical observations certified that the *A. glacialis* bloom was composed of long chain-forming, physiologically healthy cells. It is likely that, given the transience of the slack high-tide (30 min-1h), the *A.glacialis* bloom will spend only a short time in darkness, thus providing a fresh stock of cells that may enrich the bay communities at ebb tide.

An “upper-layer” community occurred in the oligohaline (1psu) freshwater tidal reach, and spread to the downstream mesohaline part of the continuum (5–18 psu) depending on species distribution ranges. Taxa diversity was higher in this part of the estuary than in the silt plug and the bay, with freshwater green algae as important contributors in terms of species number and abundance. Small centric *Cyclotella*-like diatoms predominated this “upper-layer” community. More detailed examination of this group with SEM was consistent with clone library results, confirming the existence of a great number of small thalassiosiroid taxa (mostly <10 μm), with 8 different species at least that would have escaped detection without such a thorough analysis.

Presence of some lineages (e.g. the poorly known groups of MASTs and picobiliphytes) and diversity within groups of morphologically similar taxa (e.g. cryptophytes, small centric diatoms) were estimated for the first time in the Vire estuary. Among them, the Chrysophyceae were the most noticeable. Most of these flagellates have been recorded in freshwater systems, but in lakes especially [42], [46]; here, our molecular approach revealed a large diversity of these golden algae in the Vire River sample (10 OTUs), suggesting their ecological importance in dynamic and transitional freshwater ecosystems as well. Only three OTUs were found in the Baie des Veys, but one could be an abundant member of the bay's community (most represented OTU in the BAY library).

Taxa identified by our molecular approach were widely distributed throughout the chrysophyte clades [46], including autotrophic members (e.g. *Mallomonas*) but most OTUs were affiliated to colourless bacteriovorous taxa *Paraphysomonas* spp., *Spumella* sp., and unidentified ‘*Spumella*-like’ flagellates [48]. Based on traditional microscopy, only two chrysophytes were observed along the estuarine continuum: *Mallomonas* sp. and the mixotrophic *Ollicola vangorii* (the latter was identified using SEM). The discovery of sequences in the Vire estuary (river and bay) affiliated only with environmental sequences originating from geographically distant sites (e.g. Black Sea, Arctic lakes – [61], [42]) and developing in a variety of ecosystems (fresh waters, soils, marine, or extreme environments), confirm ubiquity of the Chrysophyceae and the need for further investigation.

### 2. Morphological and molecular approaches of *in situ* phytoplankton diversity: application to estuarine waters

The traditional microscopy approach has been used as a rule for ecological studies of phytoplankton assemblages in dynamic estuaries ecosystems [62], [63], being suitable to study morphologically distinctive species and certain higher-taxon rank protistan groups [64]. Morphological identifications are unfortunately often limited to taxa previously described by cultivation-based techniques, and assaying the whole protistan community using a single microscope-based method becomes very difficult. Due to their small size, many protists may remain undetected while others are not recognized due to their cryptic nature [48], [65]. This has been clearly shown in our study, with several taxa/lineages (e.g. within chrysophytes, cryptophytes, chlorophytes) absent from our floristic list, but revealed by our molecular approach. The whole community-targeting molecular strategy, applied to three representative surface samples of the estuarine gradient, was carried out to broaden our assessment of the overall protistan diversity. In the past decade, phylogenetic analyses of the eukaryotic 18S rRNA genes have successfully described protists in a variety of aquatic environments, although such methods also include limitations, being prone to multiple sources of biases and therefore not completely reliable [10], [66]–[68]. To our knowledge, this approach has rarely been used in transitional waters such as estuarine systems [14], [15].

While providing a deeper insight into diversity of microeukaryotic communities, combination of both morphological and molecular strategies also allowed us to determine the limitations and advantages associated with each method. Our results highlight several characteristics: i) presence of chlorophytes and diatoms was lower in clone libraries than estimated by microscopy, irrespective of the location along the estuarine gradient, ii) pico- and nano-size protists were a major component in the libraries, and iii) heterotrophic lineages were clearly enhanced in the molecular approach.

The chlorophytes and diatoms lacking in our clone libraries (e.g. *Scenedesmus* spp., *Cylindrotheca closterium*) correspond to “large” species with more distinguishable features than heterotrophic flagellates or picoplankton, which explains why they are well represented in morphological approach [57]. However, most of these “easily identifiable” taxa had low densities within communities, probably leading to dilution of their sequences among the total pool of template DNA and explaining their non-detection with PCR amplification. The molecular approach was rather helpful to improve diversity estimate of the above photosynthetic lineages by recovering their smallest members that escaped our microscopical observations. Consequently, sequences related to the pico-sized *Micromonas pusilla* and *Crustomastix* (Prasinophyceae) in the bay, and others belonging to small thalassiosiroid diatoms (<10 μm) in the Vire River, were identified for the first time in this area.

The plentifulness of heterotrophic/mixotrophic small eukaryotes in the Vire River and the Baie des Veys, as revealed by the clone libraries (44% of the total OTUs retrieved), probably indicates that these communities are a major component of the microbial food web with important ecological implications in this estuarine ecosystem. The extent of their diversity and trophic interactions with phototrophic estuarine components are still poorly known and require further investigations [69]. Nevertheless, our results also corroborate similar studies highlighting that approaches based on 18S rRNA gene clone libraries using universal primers are heavily biased toward heterotrophs (e.g. alveolates and stramenopiles), to the detriment of autotrophic organisms [49]. Here, the use of microscopy partially addressed this bias and revealed the high diversity among phototrophic taxa.

The lack of congruity between morphological and molecular analyses was especially evident in the estuarine brackish surface sample (sample Cs  =  ESTUARY library). While microscopical observations led to the identification of a broad phytoplanktonic diversity in the sample including many less abundant recognizable taxa (floristic list, Table S1), the OTUs richness recovered was low because of over-representation of *Thalassiosira guillardi* related clones. The predominance of this diatom in the sample associated with the restricted sampling effort (i.e. the limited number of clones sequenced) might explain the inefficiency of our PCR-based clone library strategy. As commonly reported in previous studies (including those based on larger data sets [10], [37]), our rarefaction analyses showed that none of the clone libraries was sufficiently large to reach saturation, and therefore part of the complete protist diversity was inevitably missed. In addition to this undersampling and as discussed earlier, the non-recovery (or underrepresentation) of some taxa in clone libraries can be explained by many underlying biases, such as PCR-primer incompatibilities [66], [67], competition for primers [70] because of large variations in 18S rRNA gene copy number among taxa [68], and variable cloning efficiency. Thereby, in contrast to the microscopy-based approach, quantification of taxa based on the number of OTUs obtained with the clone library strategy is not possible. Moreover, assigning OTUs to a specific taxon rank is difficult because sequence identities vary widely with taxa considered. As suggested by other authors [10], [37], [71], we chose a 98% similarity threshold to discriminate at the genus/species level. This cut-off level associated with only partial 18S rDNA sequences provided an image of the phytoplankton diversity obviously somewhat underestimated, but for several groups, taxa discrimination was finer than with conventional microscopy, e.g. among cryptophytes (at least 4 different *Cryptomonas* species and 3 *Teleaulax*/*Plagioselmis-*like genera detected in clone libraries were missed by microscopy). Measures of protistan diversity at genotype levels are therefore difficult to compare with traditional studies relying on morphology-based taxonomic ranks [10].

Despite limitations above-mentioned, both approaches tend to be rather congruent when considering the most abundant taxa in the studied samples, as most of them, identified and counted by microscopy, were also detected by our molecular approach ( =  five of the six dominant taxa, e.g. *A. glacialis, Teleaulax*-like, many small thalassiosiroids, *R. imbricata*).

Finally, the combination of morphological and molecular approaches, both implying advantages but also limitations, were clearly complementary, providing access to greater protistan diversity than with a single method.

### 3. Conclusion

In the present study, the traditional microscopical approach allowed us to detail the changes in phytoplankton diversity occurring at a specific time along the entire estuarine gradient.

Additional 18S rDNA analysis as well as electron microscopy for three samples along the surface of the salinity gradient revealed significant additional diversity overlooked using only light microscopy and unnoticed during previous studies in the area. Several lineages such as the Chrysophyceae and *Teleaulax*-like taxa in the cryptophytes would need further characterization in the future, in terms of diversity extent (accurate identifications) and ecological implications along the estuarine continuum.

As in previous studies [23], [43], [57], our results confirm that both approaches – whether morphological or molecular – are complementary and that whatever the method, only a fraction of the whole phytoplankton community will be captured. Such combination of methods appears to be fully relevant to investigate more thoroughly the diversity of phytoplankton communities that can occur in complex ecosystems such as the transitional waters. This provides an overview of the ‘hidden’ diversity that would have escaped detection without such in-depth analysis. Our study also suggests that a significant part of the eukaryotic microbial diversity still remains to be uncovered, even in temperate estuarine waters yet considered as relatively well-known. For a molecular approach on typical estuarine samples with high diversity of low abundant taxa or predominance of few species, we suggest the use of a multi-primer PCR strategy and high-throughput sequencing, to increase the probability of detecting a broad variety of taxa and to avoid undersampling due to overrepresentation of some OTUs.

## Supporting Information

Figure S1 **Profiles of nutrient concentrations along the estuarine continuum**. Silicate, phosphate and nitrate/nitrite were analyzed in the samples collected in the water column. (A logarithmic scale was used for the data representation).
(PDF)

Figure S2 **Principal component analysis (PCA) of environmental parameters.** Samples (dots) and variables (green lines) are displayed for the first two axes. Blue dots: surface samples and red dots: samples of the near-bottom water layer.
(TIFF)

Figure S3 **Correspondence analysis (CA) based on phytoplankton composition (relative-abundance matrix).** Taxa considered in the analysis accounted for >1% of total phytoplankton in at least one sample. Blue dots: surface samples and red dots: bottom samples. *Freshwater diatoms included the following pennates: *Asterionella formosa*, *Nitzschia acicularis*, and undetermined Naviculaceae. *Chlorophytes included Chlorophyceae (*Scenedesmus* spp., *Monoraphidium contortum, Chlamydomonas* sp.) and Trebouxiophyceae (*Micractinium* sp., *Dictyosphaerium pulchellum*).
(DOC)

Figure S4 **Rarefaction curves determined for the three 18S rRNA gene libraries (VIRE River, ESTUARY and BAY).** Curves were constructed at 98% sequence similarity cut-off value.
(TIFF)

Figure S5 **Maximum likelihood (ML) tree showing the position of the non-photosynthetic stramenopiles OTUs (Bicosoecida, MASTs)**. OTUs were obtained from the the VIRE River (**V**), the ESTUARY (**Es**) and the BAY (**B**) clone libraries. Tree construction was based on an alignment of 96 partial sequences (ca 490 align positions). Number of clones per OTU is indicated in brackets. Sequences from cultured taxa appear in black, and environmental sequences in green (freshwater), blue (marine) or in brown (brackish/estuary). Bootstrap values (>50%) are indicated. The Dinophyceae *Gyrodinium fusiforme* and *Peridinium umbonatum* were used as outgroup.
(TIF)

Table S1 **Phytoplankton taxa identified by microscsopy.** Samples corresponded to surface (As → Fs) and bottom (Bb → Eb) waters. “(SEM)” refers to additional taxa identified in the surface samples **As, Cs** and **Es** (also selected for clone library analysis, referred to as VIRE River, ESTUARY and BAY) using scanning electron microscopy.
(DOC)
