# Supplementary figures and images for: Phytoplankton Diversity and Community Composition along the Estuarine Gradient of a Temperate Macrotidal Ecosystem: Combined Morphological and Molecular Approaches
Source: PLoS One. 2014 Apr 9;9(4):e94110. doi: 10.1371/journal.pone.0094110 (PMC3981767; doi:10.1371/journal.pone.0094110)

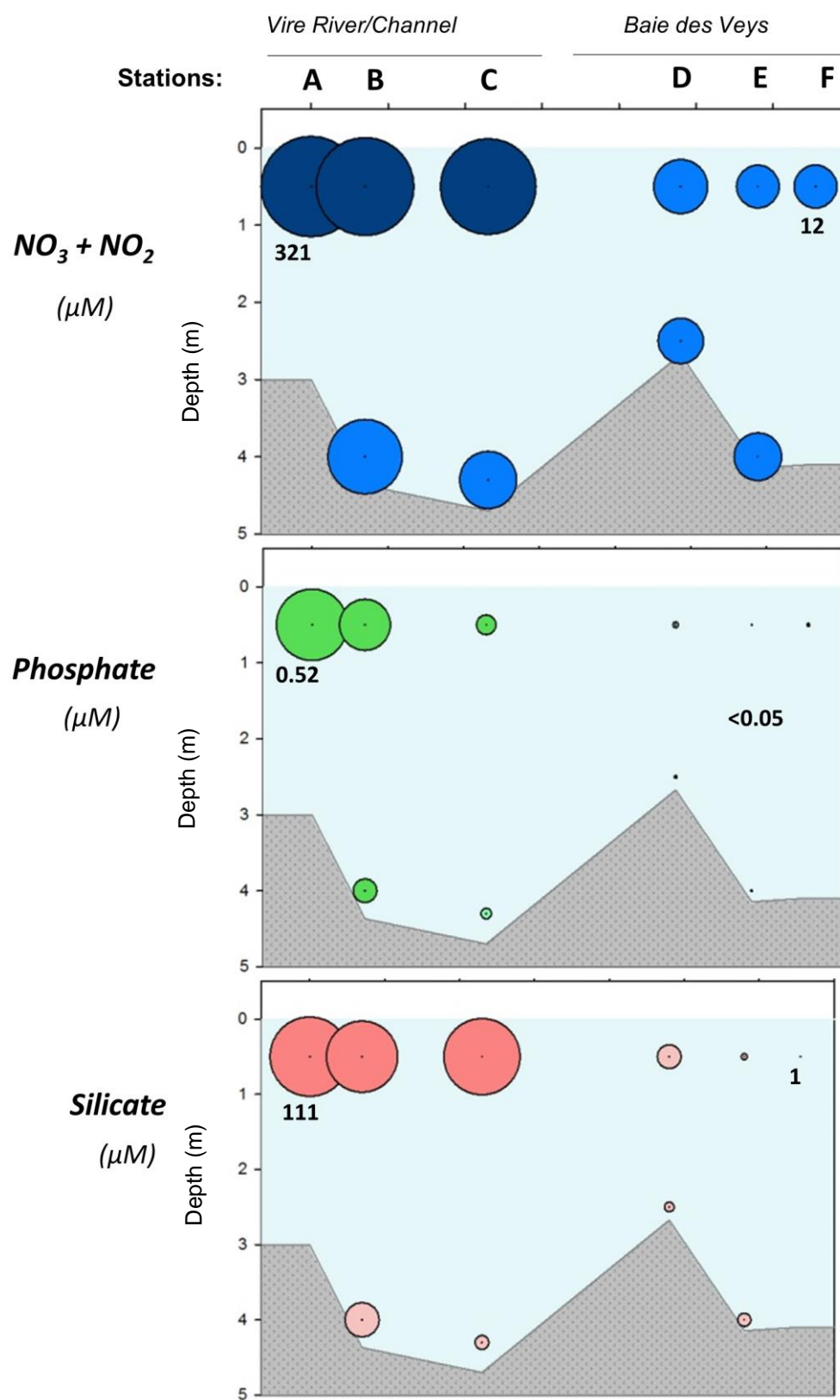

Supplement: Figure S1 — Profiles of nutrient concentrations along the estuarine continuum. Silicate, phosphate and nitrate/nitrite were analyzed in the samples collected in the water column. (A logarithmic scale was used for the data representation). (PDF) [file pone.0094110.s001.pdf]

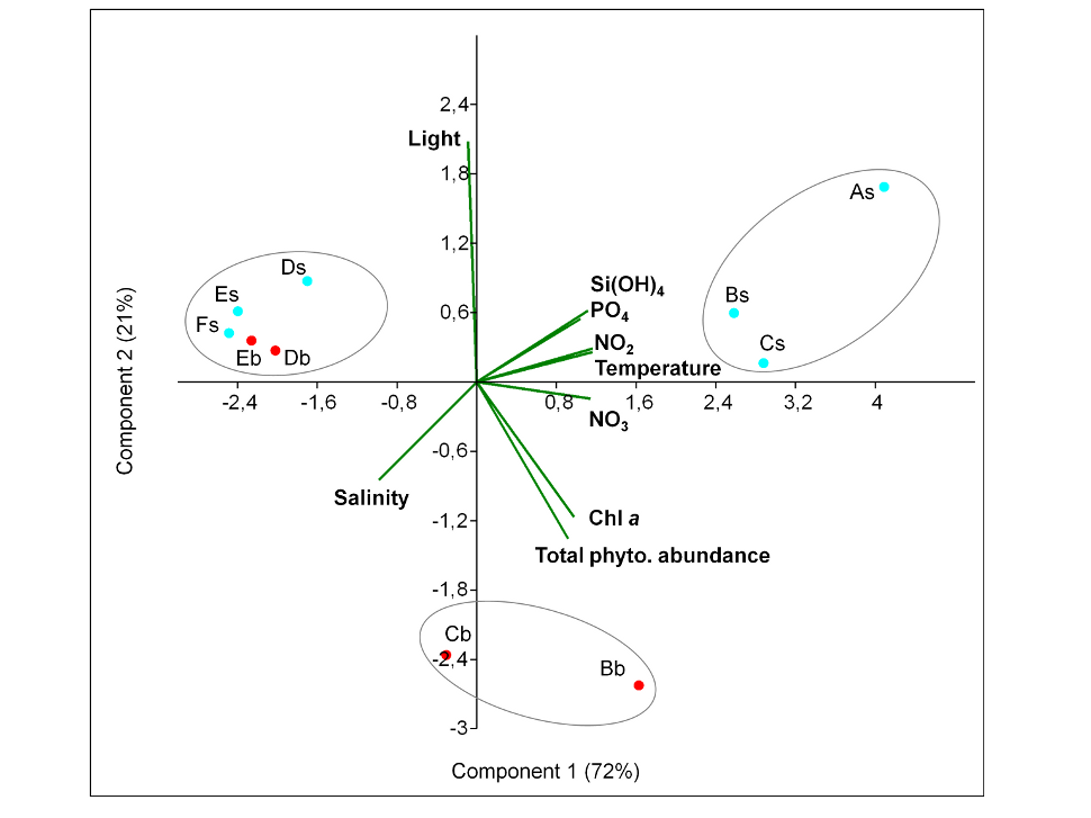

Supplement: Figure S2 — Principal component analysis (PCA) of environmental parameters. Samples (dots) and variables (green lines) are displayed for the first two axes. Blue dots: surface samples and red dots: samples of the near-bottom water layer. (TIFF) [file pone.0094110.s002.tiff]

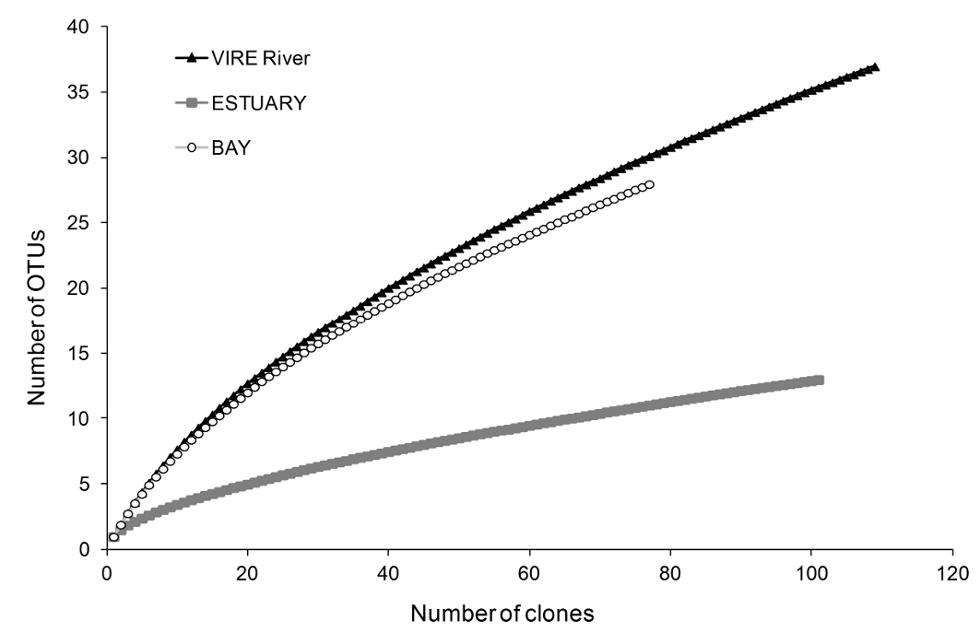

Supplement: Figure S4 — Rarefaction curves determined for the three 18S rRNA gene libraries (VIRE River, ESTUARY and BAY). Curves were constructed at 98% sequence similarity cut-off value. (TIFF) [file pone.0094110.s004.tiff]

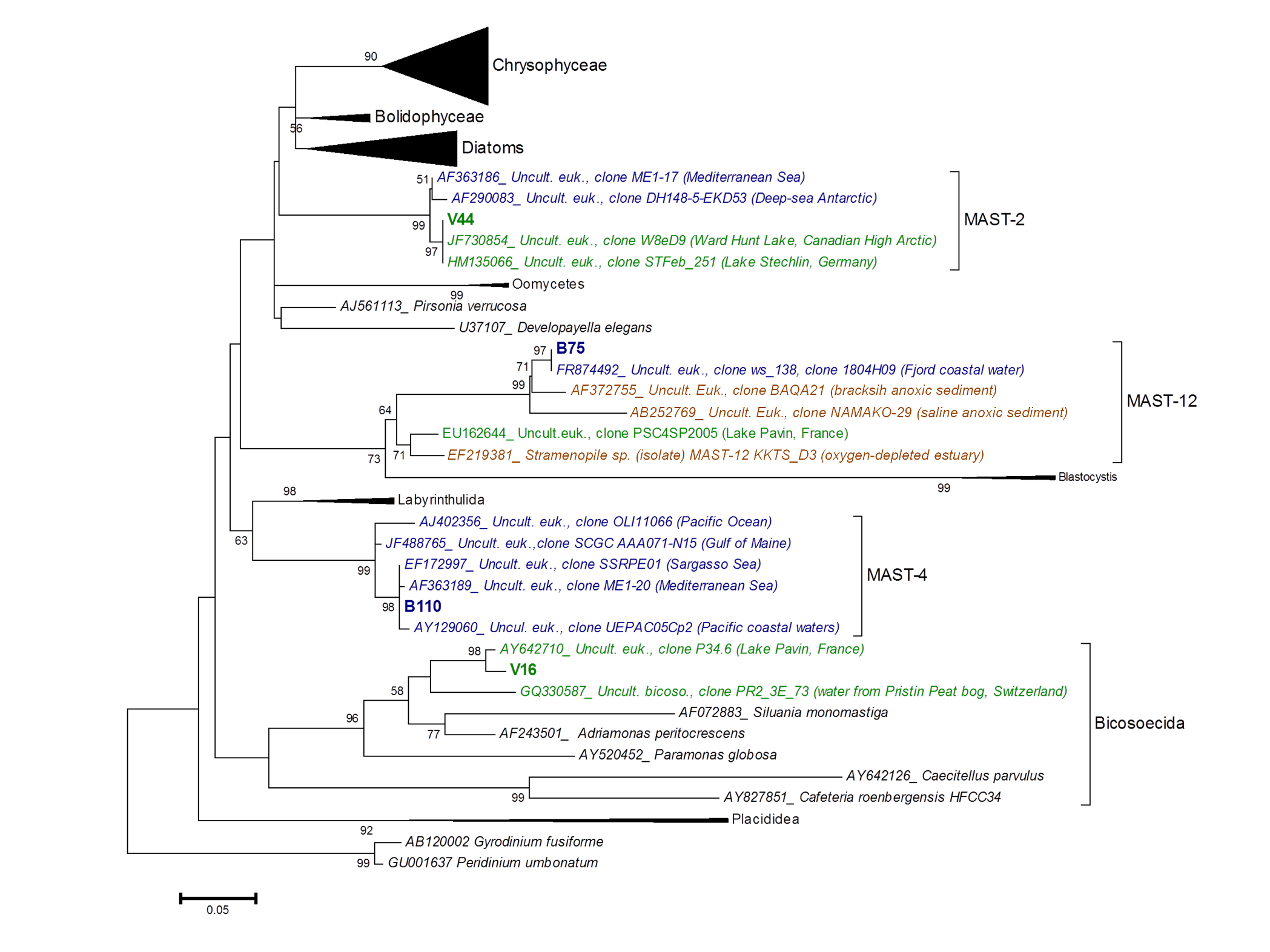

Supplement: Figure S5 — Maximum likelihood (ML) tree showing the position of the non-photosynthetic stramenopiles OTUs (Bicosoecida, MASTs). OTUs were obtained from the the VIRE River (V), the ESTUARY (Es) and the BAY (B) clone libraries. Tree construction was based on an alignment of 96 partial sequences (ca 490 align positions). Number of clones per OTU is indicated in brackets. Sequences from cultured taxa appear in black, and environmental sequences in green (freshwater), blue (marine) or in brown (brackish/estuary). Bootstrap values (>50%) are indicated. The Dinophyceae Gyrodinium fusiforme and Peridinium umbonatum were used as outgroup. (TIF) [file pone.0094110.s005.tif]
