# Supplementary material for: Phytoplankton Diversity and Community Composition along the Estuarine Gradient of a Temperate Macrotidal Ecosystem: Combined Morphological and Molecular Approaches
Source: PLoS One. 2014 Apr 9;9(4):e94110. doi: 10.1371/journal.pone.0094110 (PMC3981767; doi:10.1371/journal.pone.0094110)
Supplement: Figure S3 — Correspondence analysis (CA) based on phytoplankton composition (relative-abundance matrix). Taxa considered in the analysis accounted for >1% of total phytoplankton in at least one sample. Blue dots: surface samples and red dots: bottom samples. *Freshwater diatoms included the following pennates: Asterionella formosa, Nitzschia acicularis, and undetermined Naviculaceae. *Chlorophytes included Chlorophyceae (Scenedesmus spp., Monoraphidium contortum, Chlamydomonas sp.) and Trebouxiophyceae (Micractinium sp., Dictyosphaerium pulchellum). (DOC) [file pone.0094110.s003.doc]

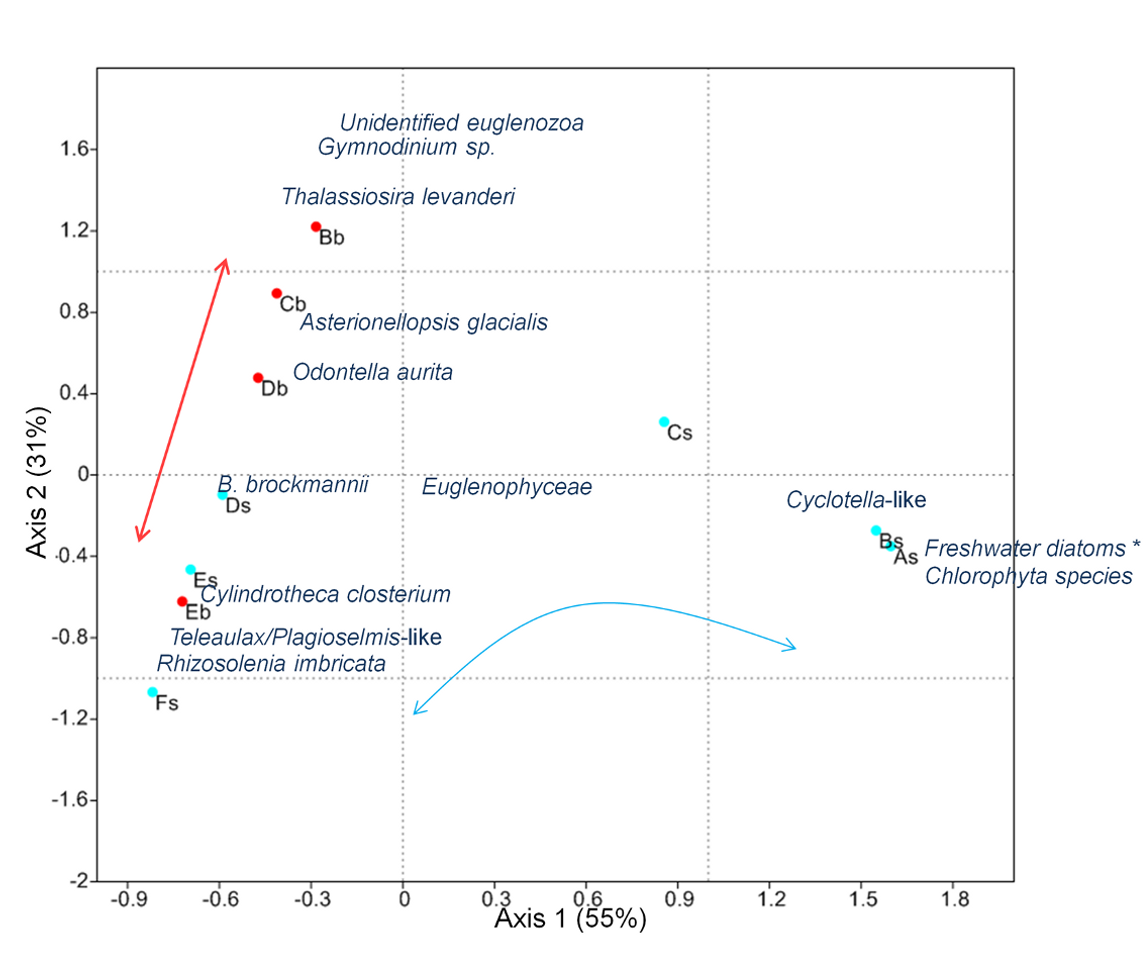


***Freshwater diatoms** include the following pennates: *Asterionella formosa*, *Nitzschia acicularis*, and undetermined Naviculaceae.

***Chlorophytes** include Chlorophyceae (*Scenedesmus* spp., *Monoraphidium contortum, Chlamydomonas* sp.) and Trebouxiophyceae (*Micractinium* sp*., Dictyosphaerium pulchellum*).
