# Supplementary material for: Phytoplankton Diversity and Community Composition along the Estuarine Gradient of a Temperate Macrotidal Ecosystem: Combined Morphological and Molecular Approaches
Source: PLoS One. 2014 Apr 9;9(4):e94110. doi: 10.1371/journal.pone.0094110 (PMC3981767; doi:10.1371/journal.pone.0094110)
Supplement: Table S1 — Phytoplankton taxa identified by microscsopy. Samples corresponded to surface (As → Fs) and bottom (Bb → Eb) waters. “(SEM)” refers to additional taxa identified in the surface samples As, Cs and Es (also selected for clone library analysis, referred to as VIRE River, ESTUARY and BAY) using scanning electron microscopy. (DOC) [file pone.0094110.s006.doc]

**Table S1**: List of phytoplankton taxa identified by microscopy, in surface (As → Fs) and bottom (Bb → Eb) water samples. “(SEM)” is noted for the additional taxa identified in the surface samples **As**, **Cs** and **Es** (also selected for clone library analysis, referred to as VIRE River, ESTUARY and BAY) using scanning electron microscopy.
